# Supplementary material for: Role of dams in reducing global flood exposure under climate change
Source: Nat Commun. 2021 Jan 18;12:417. doi: 10.1038/s41467-020-20704-0 (PMC7814128; doi:10.1038/s41467-020-20704-0)
Supplement: Supplementary file 1 — Supplementary Information [file 41467_2020_20704_MOESM1_ESM.pdf]

Role of dams in reducing global flood exposure under climate change
Supplementary Information

Julien Boulange[1*], Naota Hanasaki[1], Dai Yamazaki[2], Yadu Pokhrel[3],
[1] National Institute for Environmental Studies (NIES), Tsukuba, Japan
[2] Institute of Industrial Science, The University of Tokyo, Komaba, Tokyo, Japan
[3] Department of Civil and Environmental Engineering, Michigan State university, East Lansing, MI, USA

[*]e-mail: boulange.julien@nies.go.jp

Supplementary Note 1
Validation

The reservoir operation scheme implemented in the H08 model was developed independently of the model and is applicable to global river-routing models and runoff datasets[1]. While it has been reported to improve reservoir-release and river-discharge simulations, it is nevertheless subject to limitations, because of its simple structure. The reservoir operations are consistent with runoff data and reproduce inter-annual fluctuations in reservoir release, while monthly fluctuations are estimated from irrigation water demand[1]. The algorithm will inevitably produce some errors since actual reservoir operations are far more complex than can be simulated. Most recently developed reservoir operation schemes have attempted to capture the interactions of, and coordination between multiple reservoirs,[2,3] and to better balance human and ecosystem needs[4]. However, none has yet been deployed at a global scale, possibly due to the additional required input data. The reservoir operation scheme implemented in H08 has been assessed at 30 sites[1] and reported to reproduce observed monthly variations in reservoir storage and average monthly reservoir release.

The performance of the reservoir operation scheme was further assessed using monthly streamflow data observed at Nakhon Sawan (Thailand) used in a previous analysis by some of the authors[5]. The dataset covers the period 1956–2010. In 1964 and 1974, two major dams (the Bhumibol Dam and the Sirikit Dam) officially began operation. Monthly discharges were therefore divided into two sets, pre- and post-dam (before 1964 and after 1974, discarding the period between 1964 and 1974), matching our model simulation setup. The observed and simulated discharges before and after dam implementation are presented in Supplementary Figure S13.

Leveraging a previous study[6] in which the effects of dams on flood occurrence in the US were investigated, and limiting our analysis to large dams in the GranD database with catchment areas larger than 2,000 km^2, discharges before and after dam implementation were assessed in 10 locations in the US (Supplementary Table S3). All streamflow observations were retrieved from the USGS website and separated into two periods: before and after dam construction (Table S3). We then overlaid our simulations using the appropriate experiment (with or without dam implementation).

The simulations tended to faithfully reproduce monthly variations in discharge for simulations with and without dams (Figs. S14 to S23). In the simulations with dams, peak discharge decreased while low discharge increased. These dams therefore decreased the seasonality of discharge. According to the reservoir operation scheme, none of the dams was operated to minimize flood risk (See Supplementary Material S2).

Next, we assessed how simulated maximum peak streamflows compared with the largest past streamflows ever observed at 33 locations (Supplementary Table S4). All observations were obtained from a USGS report[7] and compared separately to maximum daily streamflows in the historical simulation for the four GCMs. Generally, forcing the coupled model with four GCMs resulted in maximum daily streamflows of the same magnitude (see Supplementary Fig. S24). The simulated maximum daily streamflows were usually lower than those reported in the literature for a given catchment. In contrast, the simulated maximum daily streamflows for a few catchments were higher than those ever recorded (for example, for the Niger and the Ganges). The non-consideration of infiltration and evaporation processes in rivers may explain such discrepancies, particularly in hot and (semi-)arid climate zones (see Fig. S4)[8]. The presence of a large lake on the St. Lawrence River also compromised the reproduction of peak discharge (see Fig. S24 and Table S4). Overall, these results were similar to those reported in the original validation of the CaMa-Flood model[8].

Supplementary Note 2
Role of dams

When properly operated, dams are reported to mitigate downstream flooding[9]. For example, in 2011, a massive flood occurred in central Thailand despite two major dams (the Bhumibol and Sirikit) that collectively store about 10,000,000,000 m^3[ref 10]. Similarly, in Japan, two dams (the Miyagase and Shiroyama) that collectively stored 72,000,000 m^3 of water were reported to have prevented an additional 1.1 m of flooding-depth downstream during typhoon Hagibis in October 2019 (https://www.ktr.mlit.go.jp/ktr_content/content/000760679.pdf; report produced by the Ministry of Land, Infrastructure, Transport and Tourism (Japan); in Japanese).

Although alternative reservoir operations may provide additional benefits against flood risk (e.g. by lowering flooding depth and flooded area)[5], it has been reported that prioritizing water security is necessary to (at least partially) offset the effects of climate change, although potentially at the cost of more effective flood mitigation[11].

When mismanaged, dams can increase the risk of flooding, and unexpected accidents may cause fatal disasters downstream. There are approximately 14,000 dams in the US classified as "high hazard potential," indicating that any misoperation would likely result in loss of life[12,13]. In India, improper dam release caused approximately 600 deaths and the displacement of 309,250 people between 2008 and 2011. More generally, many fatalities have been caused by the release of water from dams without relevant downstream information[14].

Globally, the failure of 70 large dams (> 15 m in height) as a result of floods has been reported. Among those, 15 failures caused a total of over 10,000 fatalities[14]. While many dams built in the US before 1930 have failed, often with few or no fatalities, dam failures between 1970 and 1980 resulted in about 500 deaths and $2 billion worth of losses and damage[15]. Dam failure was listed in the Dartmouth database as the main cause of flooding for nine events, in the US, Australia, and Africa. These events were generally associated with around 9,800 displaced people.

Factors that may contribute to dam failure include internal erosion in dams and their foundations[16], overtopping[17], and earthquake[18], among other[19]. Embankment dams failure has mostly been attributed to overtopping (41.0%) and poor construction materials (41.5%)[20,21]. Aging dam infrastructure (mainly in developing countries), combined with a changing climate, may increase the likelihood of dam failure in the future[13].

Supplementary Note 3

Comparison with global and regional studies

The impact of dams on floods

While lakes and reservoirs affect the severity of flooding by modulating the timing and magnitude of streamflow, neither has been physically considered in global flood studies. Some recent studies have, however included dams in flood studies at the regional and national scales.

In the contiguous US, the alteration of flood flows by dams has been reported in every region. It is particularly severe west of the Mississippi, in the southern Great Plains, and in northern California[22]. These findings were confirmed by a study that employed a spatially distributed hydrological model[23] which found that the effect of reservoirs on extreme high flows was substantial and resulted in significant differences in 5-year return levels before and after dam implementation. Few dams increased the likelihood of flooding compared with the no-dam simulation, which could occur due to excessive overflow (Fig. 1 and Fig. S5c). Nevertheless, dams strongly decreased the occurrence of the 5-year return period and, consistent with this study, the effect of dams on the 5-year return period decreased progressively, becoming relatively small (or negligible) at the river mouth (Fig. 1, Fig. S5c).

A recent study employed a Random Forest procedure in the CONUS region to capture up and downstream differences in the 100-year return period flow[24]. This methodology was reported to significantly improve the accuracy of flood hazard maps and could be quantified by the flood attenuation index (FAI). There was no discernible pattern in the spatial distribution of the FAI, reportedly due to the heterogeneity of the dam characteristics. This is again similar to the reduction in future flood frequencies observed in this study along major sections of rivers containing multiple high-capacity dams (Figs. 1 and S5c). Almost half the dams (47%) were classified as having a high FAI, which decreased flood peak by at least 60%[24]. Analogous to the first study[22], these dams were located in the Pacific Northwest, Lower Colorado basin, and lower Mississippi. Since no reservoir operation scheme was involved, the dams did not increase the risk of flooding.

Population exposure to floods

Few studies have reported on the exposure of global populations to present and future floods, with some authors assessing only exposure to historical 100-year floods (as in this study). In contrast, global exposure to all floods has been reported in other analyses.

On average, 5.6 ± 2.3 million people have historically been exposed to a 100-year flood[25], relatively close to our estimate of 9.2 million (see Table S2). Jongman et al. (ref [26]) estimated that, at most, the number of people worldwide exposed to a 100-year river flood amounts to 805 million. By 2050, that number will reach 1.05 billion[26], depending on the extent of global warming and population growth. Fixing populations level at the 2005 estimate, it has been reported that 27, 62, or 93 million people would be exposed to global floods under global warming conditions of +2°C, +4°C and +6°C, respectively[25]. These estimates can be compared with our projections of 31.5 million people (for RCP2.6) or 55.2 million people (RCP6.0) who will be exposed to a historical 100-year flood by the end of the 21st century (see Table S2).

Jonkman[27] concluded that the 6,297 flood disasters documented from 1975 to 2001 affected over four billion people and resulted in 1.99 million fatalities. Using computer models, the number of people exposed to floods worldwide was estimated to be 54 million[28], 58 million[29], and 123.4 million[30]. Estimates of future global human exposure to floods are limited by the accuracy of input datasets, such as those for population growth and climate projections. Nevertheless, the number of people exposed to a doubling of flood frequency ranges from 323 million to 570 million people, depending on the emission scenario[31]. At 3°C warming, the global population exposed to flooding could be between 90–186 million people

under a fossil-fueled development scenario (SSP5) or 113–241 million people in a regional rivalry scenario (SSP3)[29].

It is important to note that the future population distributions used in this study were based on assumptions about future fertility, mortality, migration, and educational transitions that are consistent with the SSP storylines[32]. Jones et al.[33] asserted that the most influential assumptions are related to changes in national-level populations, the rate of urbanization, and the spatial style of development. Neither sea-level rise nor increases in flood occurrence was taken into consideration in deriving these future population demographics. In the latest global population distribution projection by Vollset et al.[34] , although economic and geopolitical effects were accounted for, the effects of flooding were not considered.

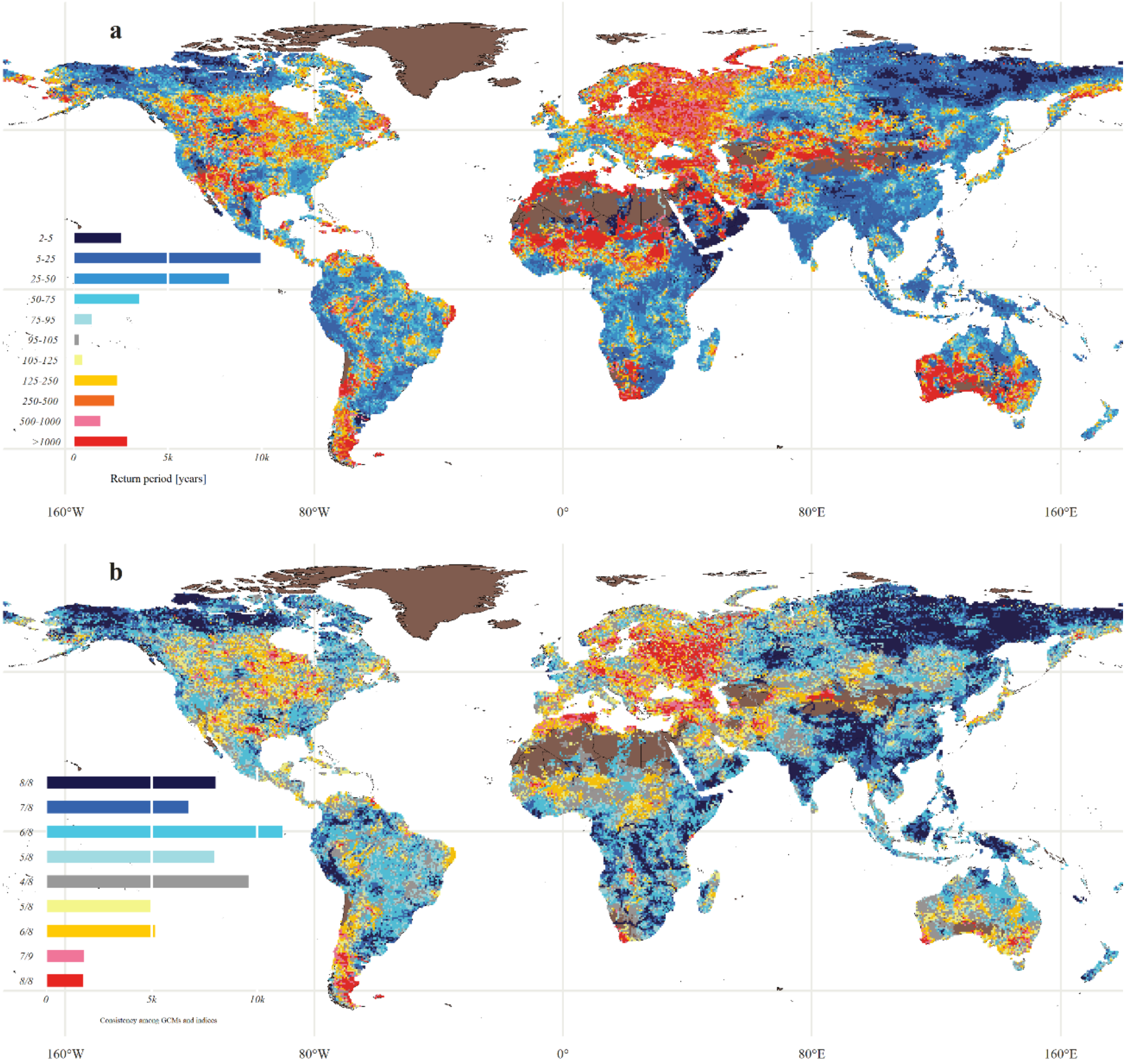

Supplementary Figure 1: Frequency of historically 100-year flood in the future. a, multi-model median return period (years) in 21C for discharge corresponding to the historical 100-year flood for the no dam experiment and RCP6.0. **b**, Consistency in the model projected change in historical 100-year discharge given 4 GCMs and 2 extreme discharges indices (no dam experiment and RCP6.0).

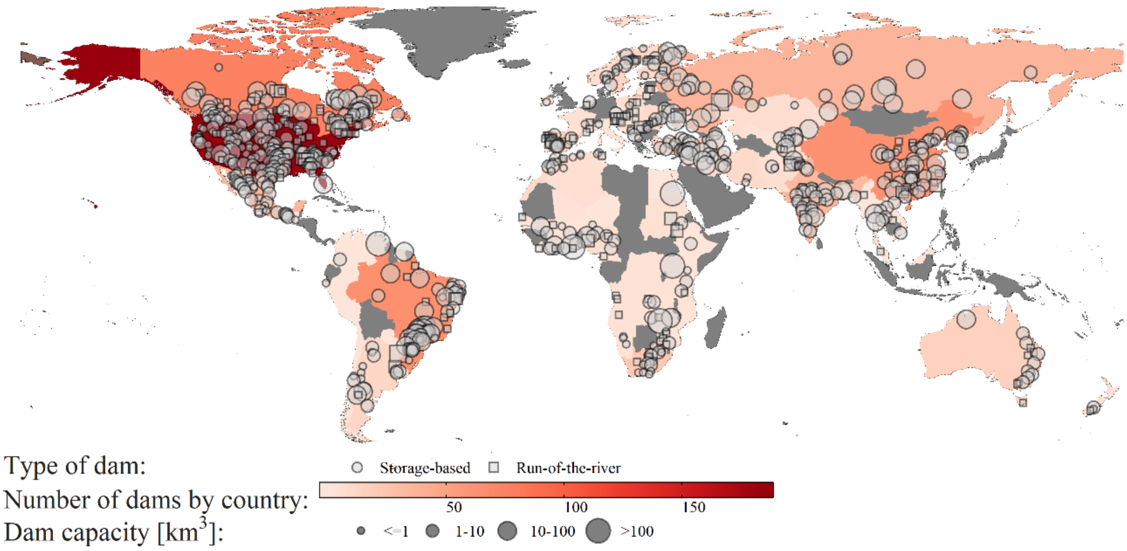

Supplementary Figure 2: Locations of the global dams included in this study. Note: The run-of-the-river dams consisted of dams which storage divided by the annual mean inflow was inferior or equal to 0.05 year^{-1}. Storage-based dams simply consist of all other remaining dams.

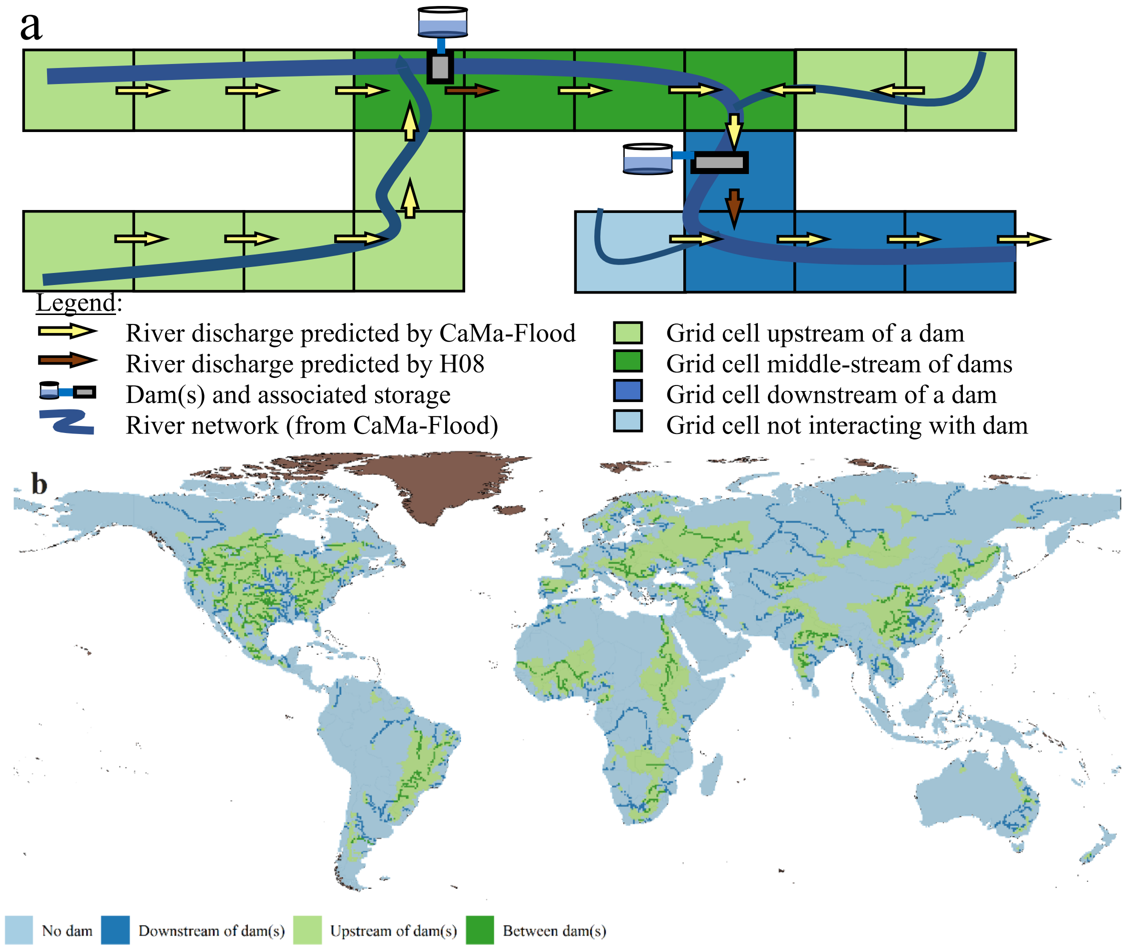

Supplementary Figure 3: Details of the coupling procedure between H08 and CaMa-Flood. a, Schematics of the coupled H08 and CaMa-Flood models for a hypothetical river. **b**, Global distribution of the position of grid cells relative to dams.

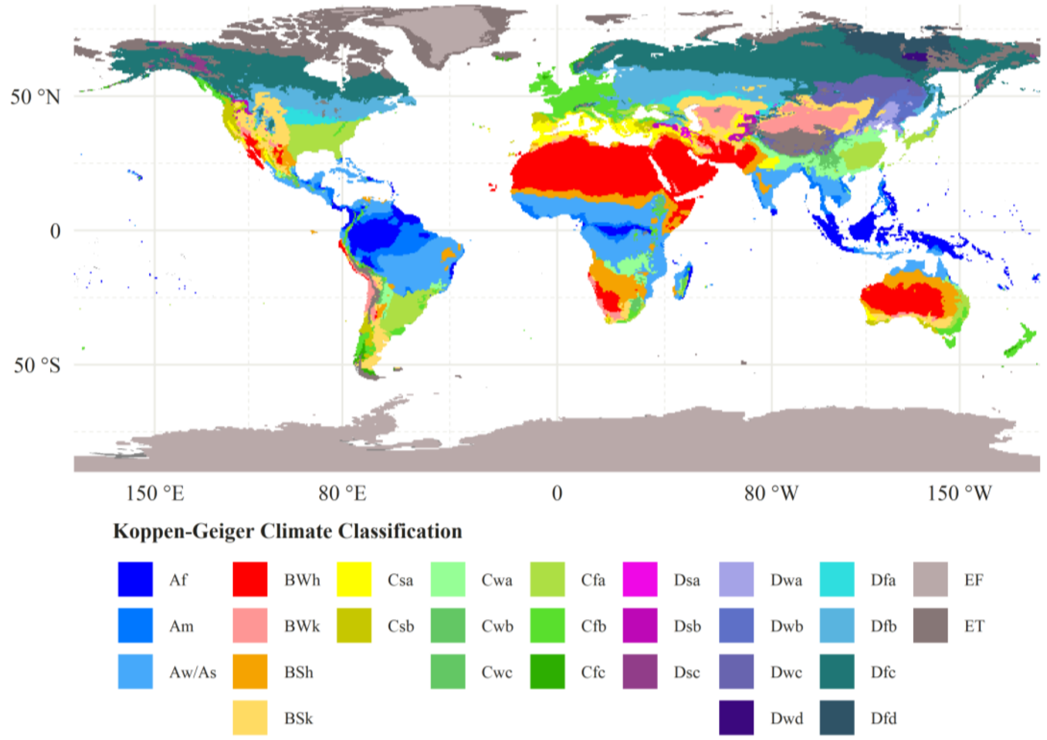

Supplementary Figure 4: Köppen-Geiger climate classification. The main five groups are A (tropical), B (dry), C (temperate), D (continental), and E (polar).

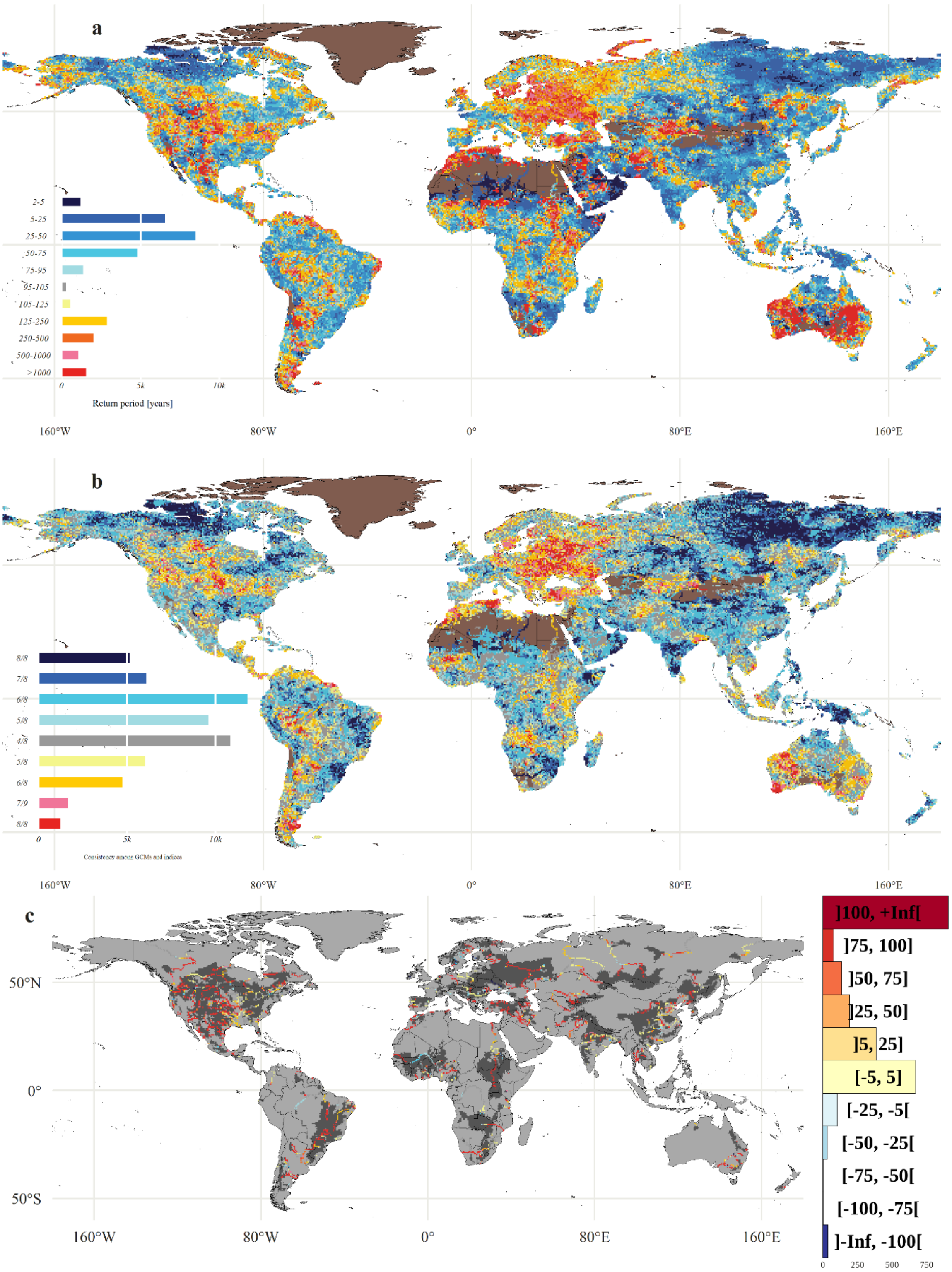

Supplementary Figure 5: Future floods in the RCP2.6 scenario. a, b, Identical to Supplementary Figure 1a and b, but for RCP2.6. Panel **c** is identical to Fig. 1 but for RCP2.6.

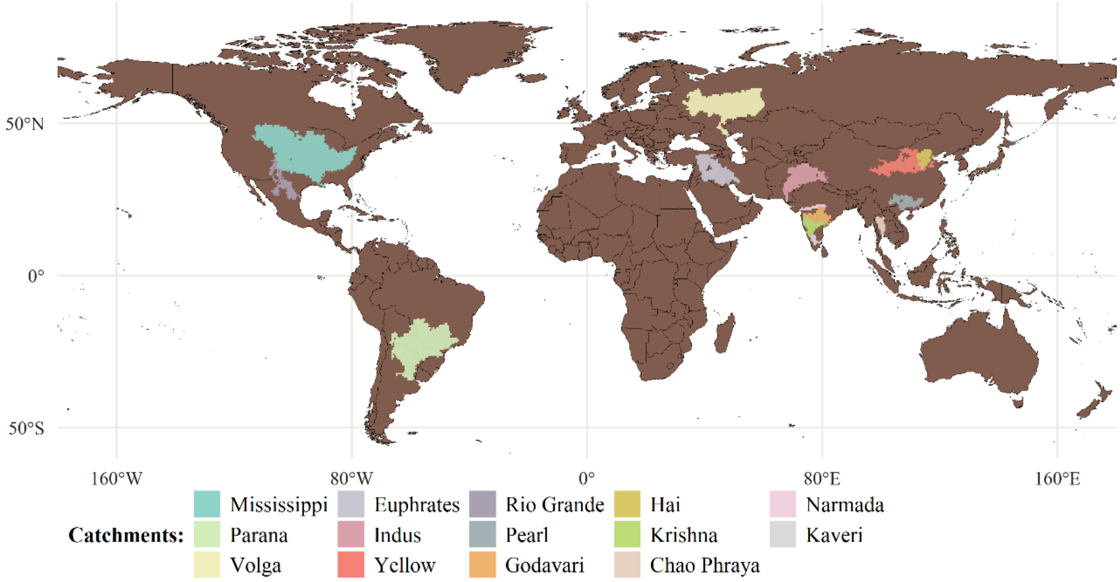

Supplementary Figure 6: Locations of the 14 catchments considered in this study.

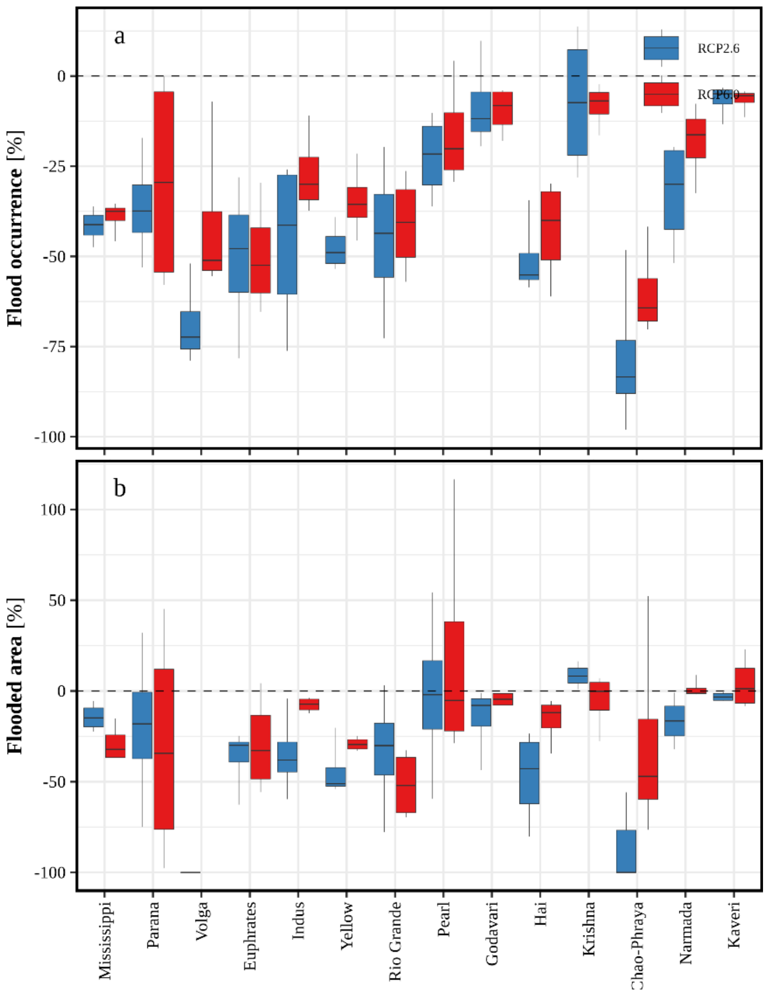

Supplementary Figure 7: Same as Figure 3 but using P_{05} to identify flood events.

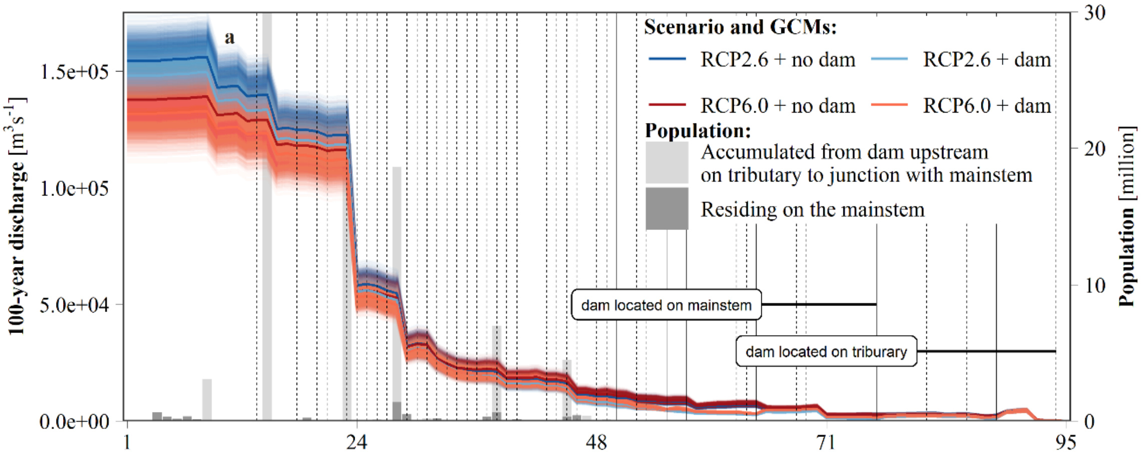

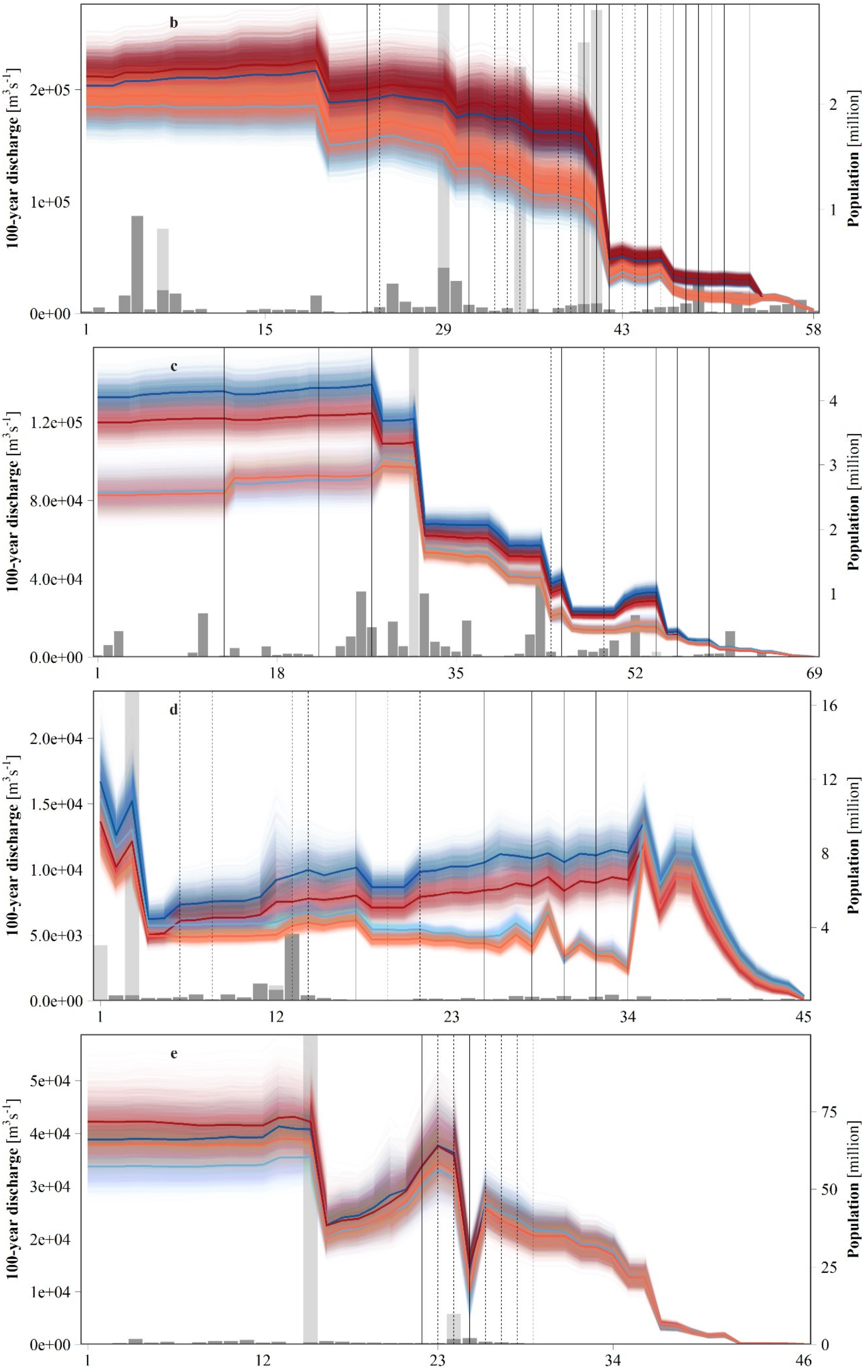
b
c
d
e
100-year discharge [m^{3}s^{-1}]
Population [million]
2e+05
1e+05
0e+00
1.2e+05
8.0e+04
4.0e+04
0.0e+00
2.0e+04
1.5e+04
1.0e+04
5.0e+03
0.0e+00
5e+04
4e+04
3e+04
2e+04
1e+04
0e+00
1
15
29
43
58
18
35
52
69
12
23
34
45
46
2
4
3
16
8
75
50
25
0

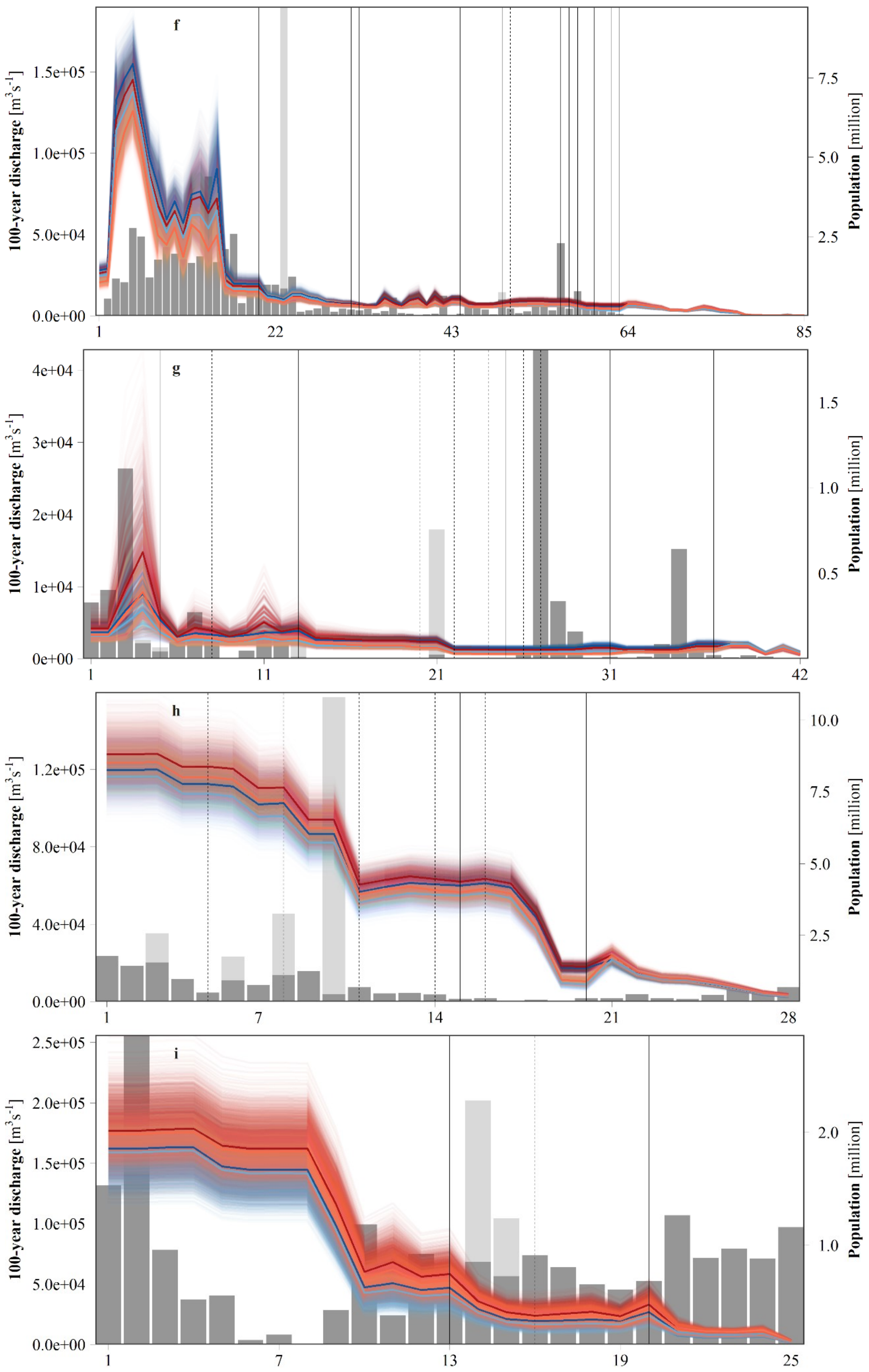

f
g
h
i
100-year discharge [m^{3}s^{-1}]
Population [million]
1.5e+05
1.0e+05
5.0e+04
0.0e+00
7.5
5.0
2.5
1
22
43
64
85
4e+04
3e+04
2e+04
1e+04
0e+00
1.5
1.0
0.5
11
21
31
42
1.2e+05
8.0e+04
4.0e+04
10.0
7
14
28
2.5e+05
2.0e+05
1.0e+05
2.0
13
19
25

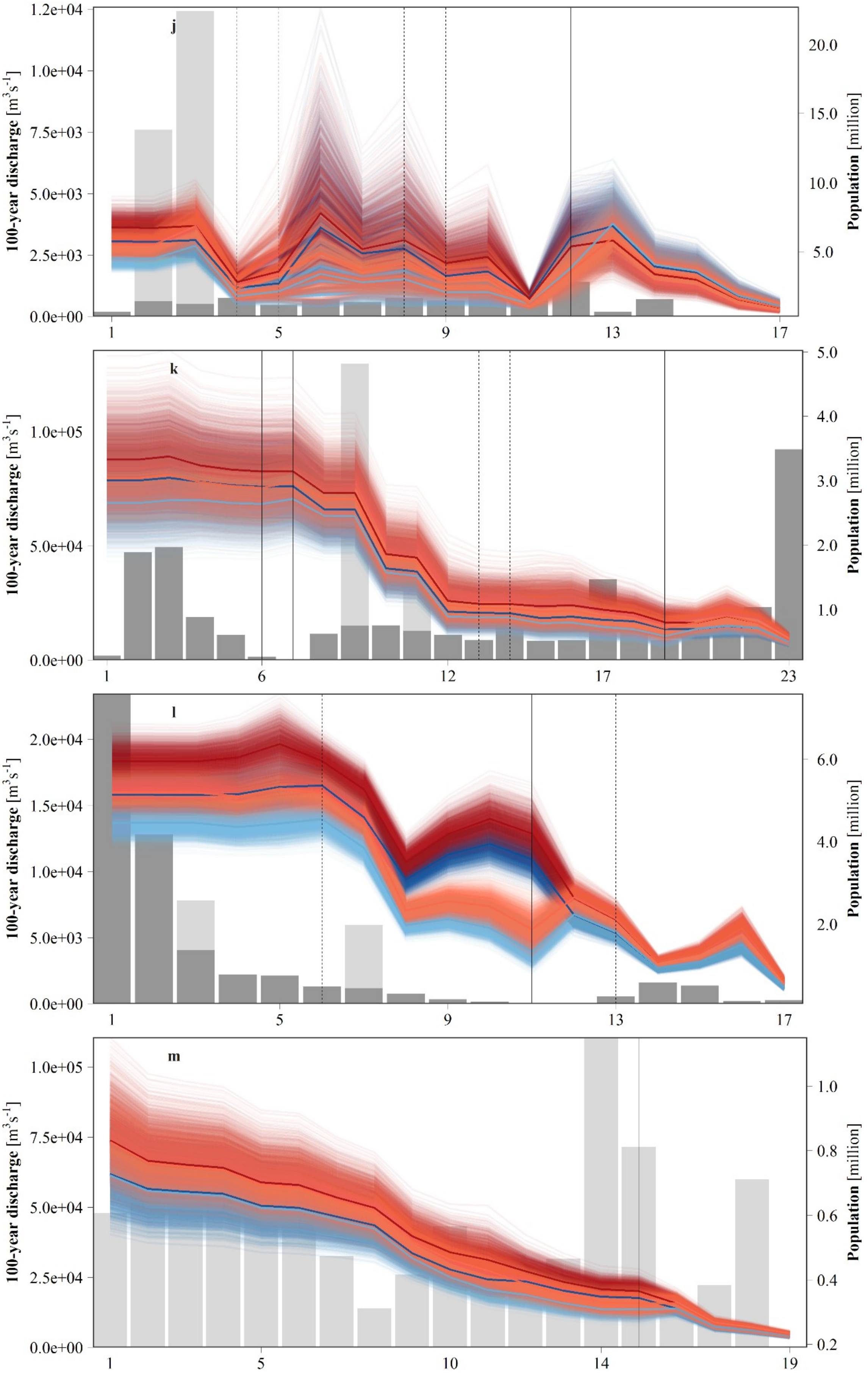
j
k
l
m
100-year discharge [m3s-1]
Population [million]
1.2e+04
1.0e+04
7.5e+03
5.0e+03
2.5e+03
0.0e+00
20.0
15.0
10.0
5.0
1 5 9 13 17
1.0e+05
5.0e+04
0.0e+00
5.0
4.0
3.0
2.0
1.0
1 6 12 17 23
2.0e+04
1.5e+04
1.0e+04
5.0e+03
0.0e+00
6.0
4.0
2.0
1 5 9 13 17
1.0e+05
7.5e+04
5.0e+04
2.5e+04
0.0e+00
1.0
0.8
0.6
0.4
0.2
1 5 10 14 19

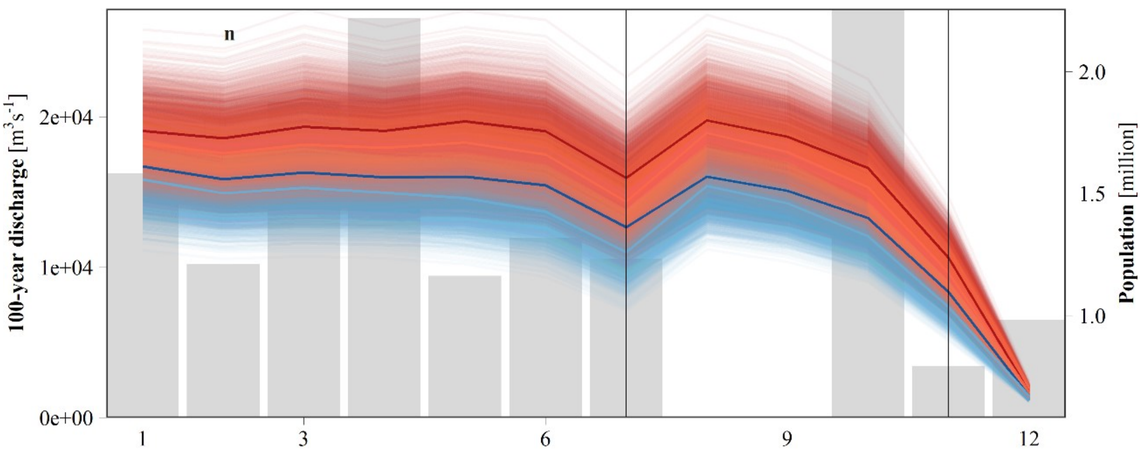

Supplementary Figure 8: Mean and every 1000 bootstrap iterations of the 100-year river discharge for the future simulation (2070-2099) along the mainstem of a river, from the headwater to the river mouth (x=1) for two RCPs and two experiments (with and without dams) for 14 catchments. a, Mississippi, **b** Paraná, **c** Volga, **d** Euphrates, **e** Indus, **f** Yellow, **g** Rio Grande, **h** Pearl, **i** Godavari, **j** Hai, **k** Krishna, **l** Chao-Phraya, **m** Narmada, **n** Kaveri. Note: A bootstrap methodology (1000 iteration, see Methods) was employed for fitting the extreme discharge to a Gumbel distribution. All 100-year extreme discharge [m^3 s^{-1}] are reported along the mainstem of 14 catchments for 2GCMs and 2 experiments, giving a better sense of uncertainty encompassed in our prediction. The vertical lines indicate the presence of a dam on the mainstem (plain line) or tributary (broken line). When dams are located on the main channel, the vertical line indicates the grid cell where it is located. For dams located on tributaries, the broken vertical line is placed at the grid cell where the tributary joins with the main stem. The population residing on the main channel is indicated in dark grey bars. The population living below a dam on a tributary is cumulatively added until the tributary joins with the main channel and it is displayed at the junction point as a light grey bar.

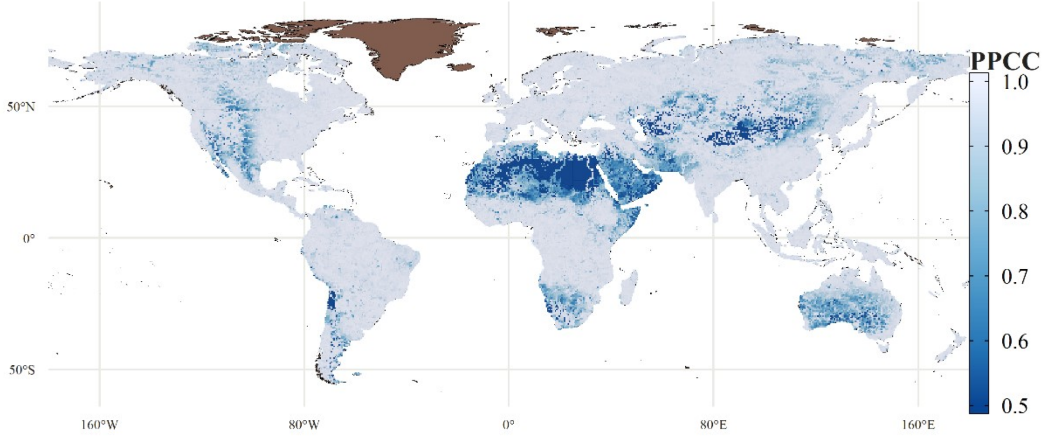

Supplementary Figure 9: Average probability plot correlation coefficient (PPCC) statistic across the 4 GCMs for the historical simulation. Note: A PPCC score close to 1 indicates that the distribution of the extreme series is well fitted by the Gumbel distribution. For a sample size of 30, the critical PPCC score at the 95th level of significance was reported43 to be approximately 0.96 (See Methods).

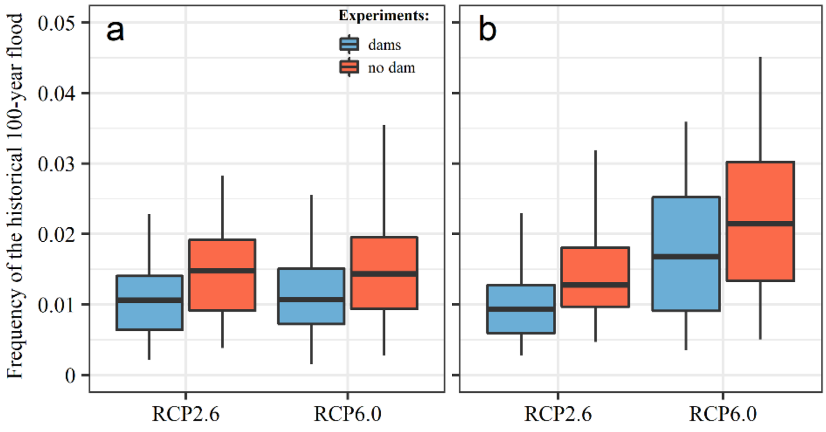

Supplementary Figure 10: Frequency of the historical 100-year flood in the future. Given the experiments considering and not considering dams: a, Evolution compared to the historical period for mid-century and b, Evolution compared to the historical period for late century.

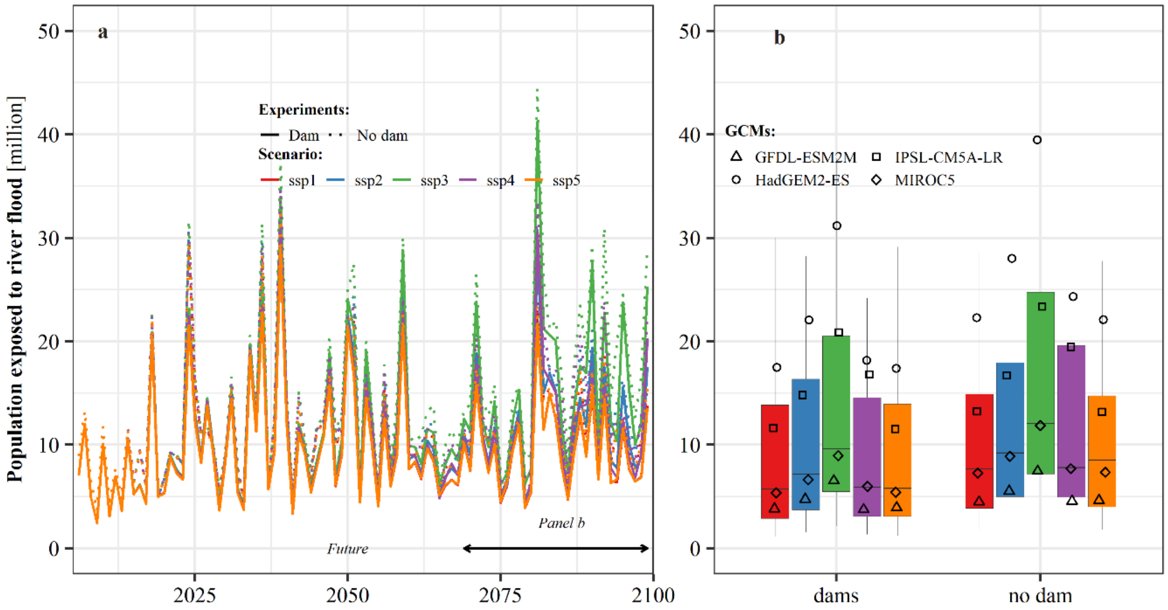

Supplementary Figure 11: Population exposure to the historical 100-year river flood for various population projections. **a,** GCM averages of the population living below dams exposed to the historical 100-year river flood for future simulations for RCP2.6, dam and no dam experiments (line types) and the 5 ssps (colors). **b**, The 95th and 5th range (whiskers), median (horizontal lines in each bar), and 1st and 3rd quartiles (height of box) and individual mean values among GCMs (markers) of the population exposed to the historical 100-year flood for grid-cells located below dams over the 2070-2099 period.

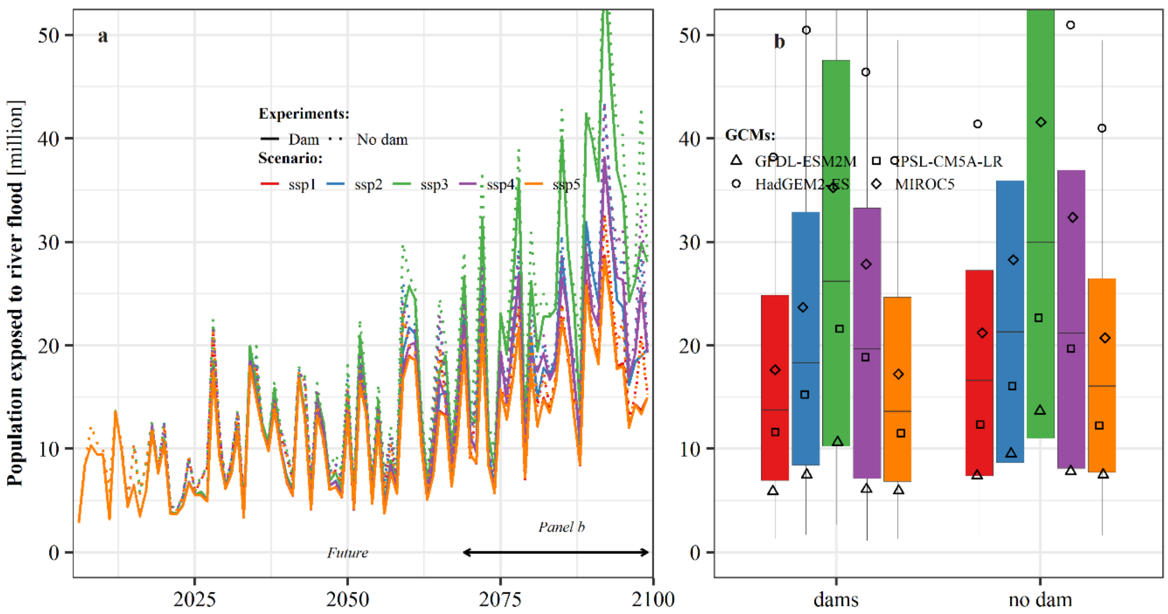

Supplementary Figure 12: Identical to Supplementary Figure 11, but for RCP6.0.

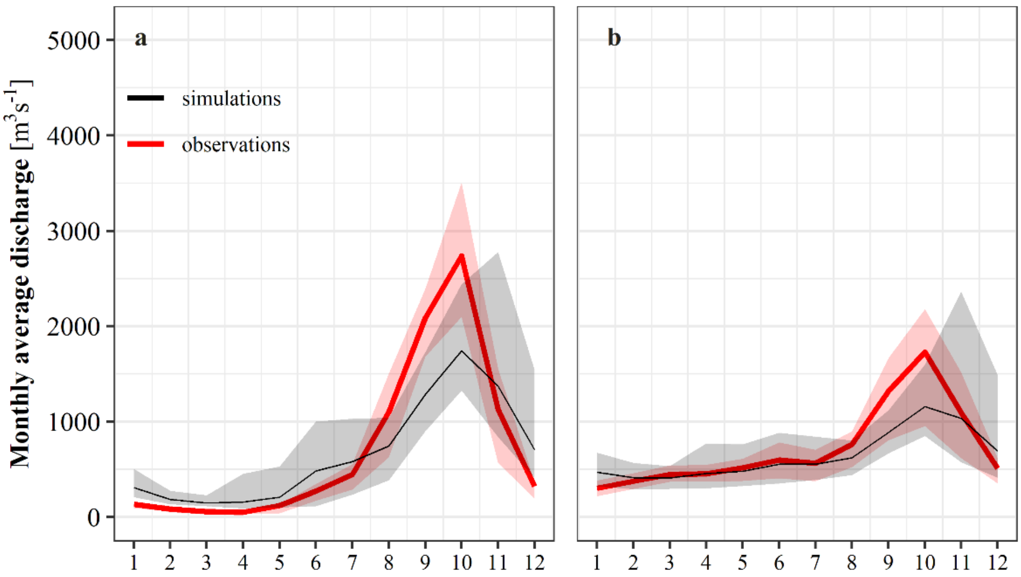

Supplementary Figure 13: Observed and simulated monthly discharges at Nakhon Sawan (Thailand). (**a**) before 1964 and (**b**) after 1974. The Bhumibol and Sirikit dams (catchment area: 26, 400 km^2 and 13,130 km^2, respectively) started operation on 1964 and 1974, respectively. Consequently, panel **a** is analogue to the simulation with no dam implementation while panel **b** represents the dam implementation scenario. The color bands show the 95th intervals associated with the observed (red) and simulated (black) monthly average discharge.

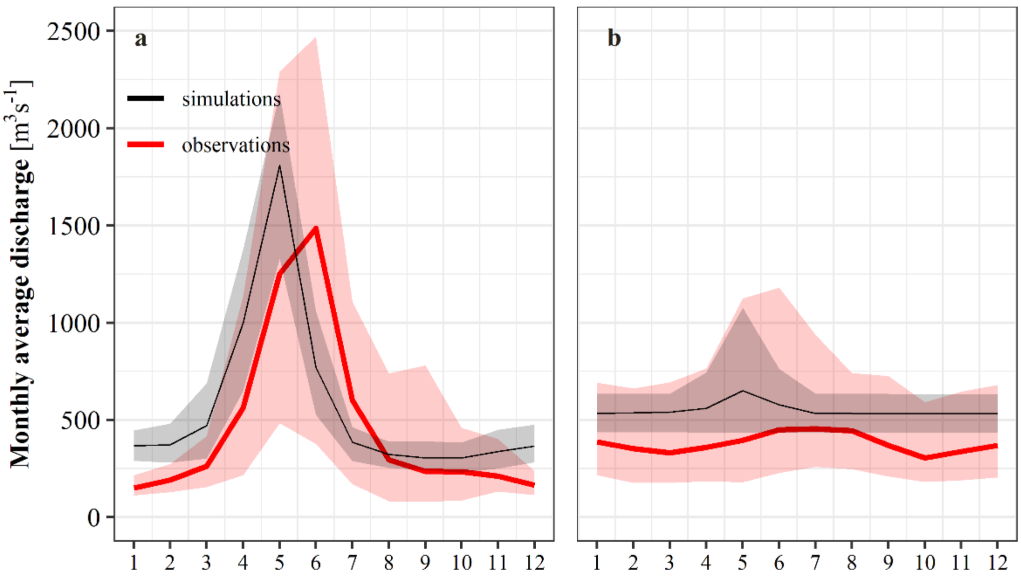

Supplementary Figure 14: **Observed and simulated monthly discharges at Glenn Canyon dam (Colorado River, US)**. (**a**) before 1963 and (**b**) after 1963 when the dam entered active operation. The color bands show the 95th intervals associated with the observed (red) and simulated (black) monthly average discharge.

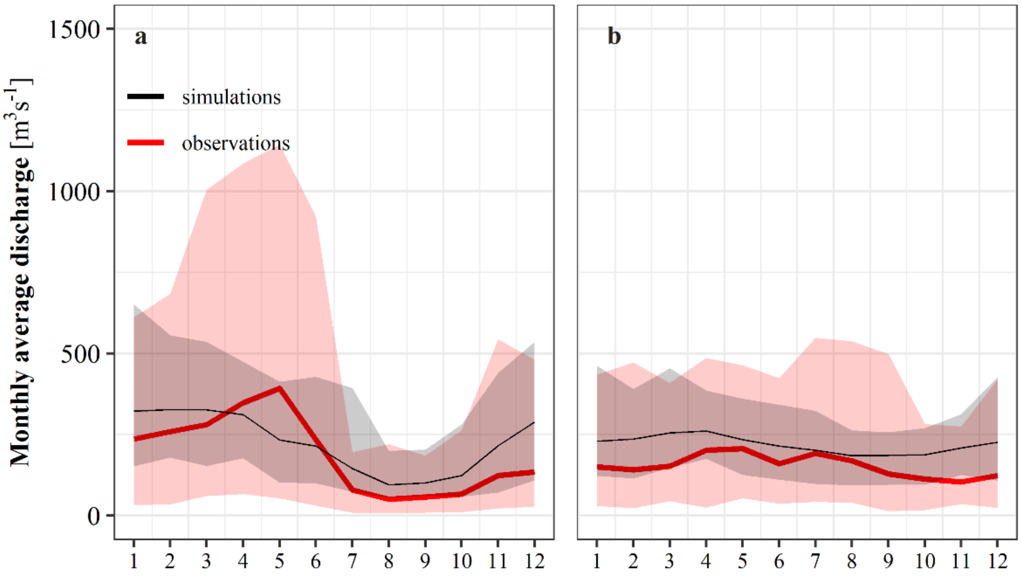

Supplementary Figure 15: Observed and simulated monthly discharges at Bull Shoals dam (US). (**a**) before 1951 and (**b**) after 1951 when the dam entered active operation. The color bands show the 95th intervals associated with the observed (red) and simulated (black) monthly average discharge.

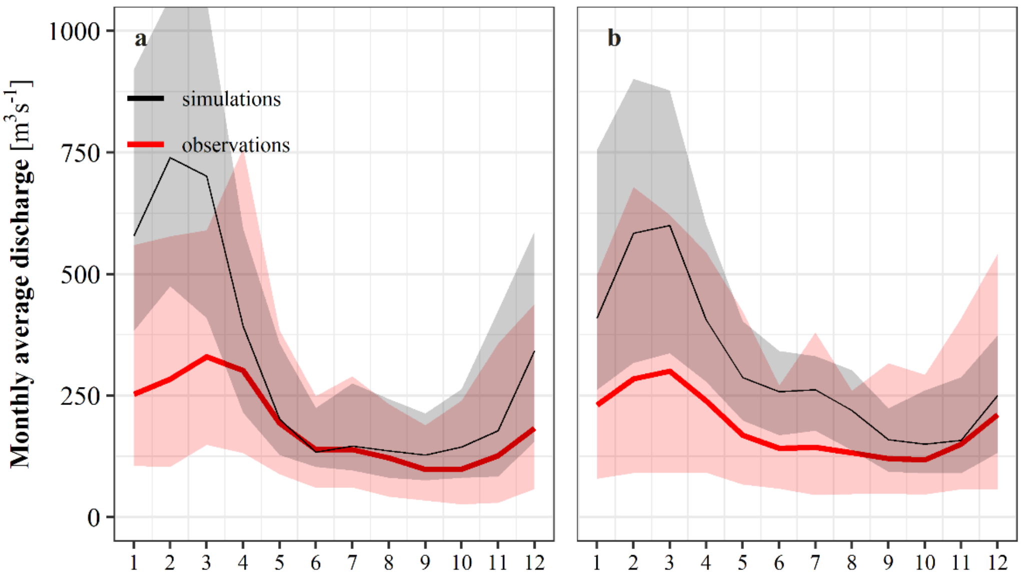

Supplementary Figure 16: Observed and simulated monthly discharges downstream of West point dam (US). (**a**) before 1974 and (**b**) after 1974 when the dam entered active operation. The color bands show the 95th intervals associated with the observed (red) and simulated (black) monthly average discharge.

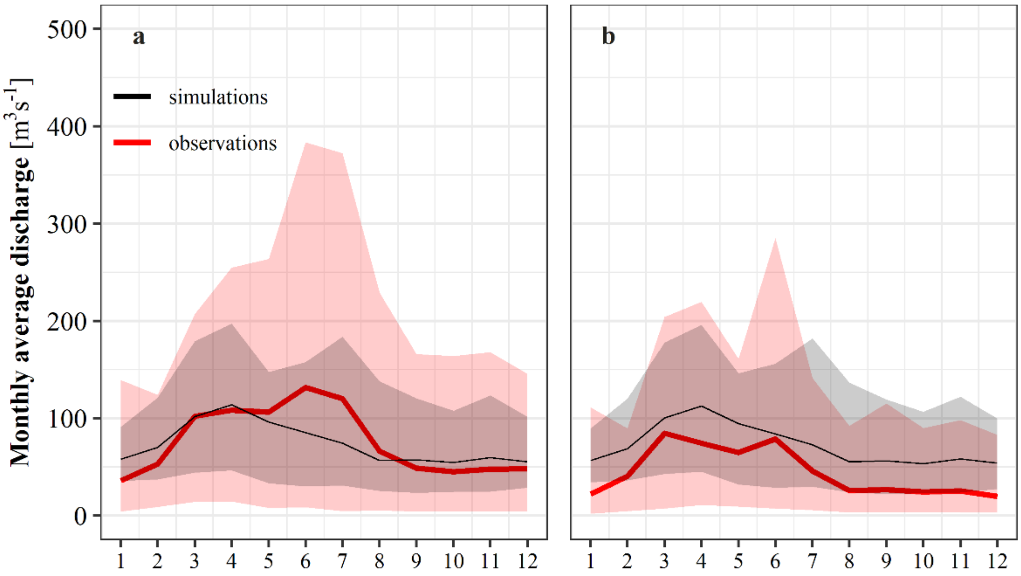

Supplementary Figure 17: Observed and simulated monthly discharges downstream of Coralville dam (US). (**a**) before 1958 and (**b**) after 1958 when the dam entered active operation. The color bands show the 95th intervals associated with the observed (red) and simulated (black) monthly average discharge.

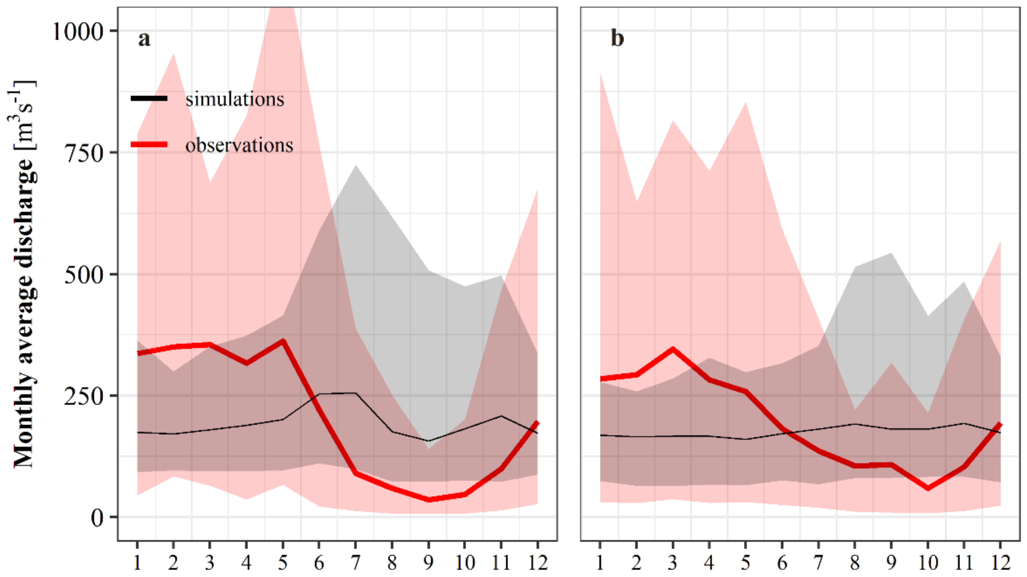

Supplementary Figure 18: Observed and simulated monthly discharges downstream of Iron Bridge dam (US). (**a**) before 1960 and (**b**) after 1960 when the dam entered active operation. The color bands show the 95th intervals associated with the observed (red) and simulated (black) monthly average discharge.

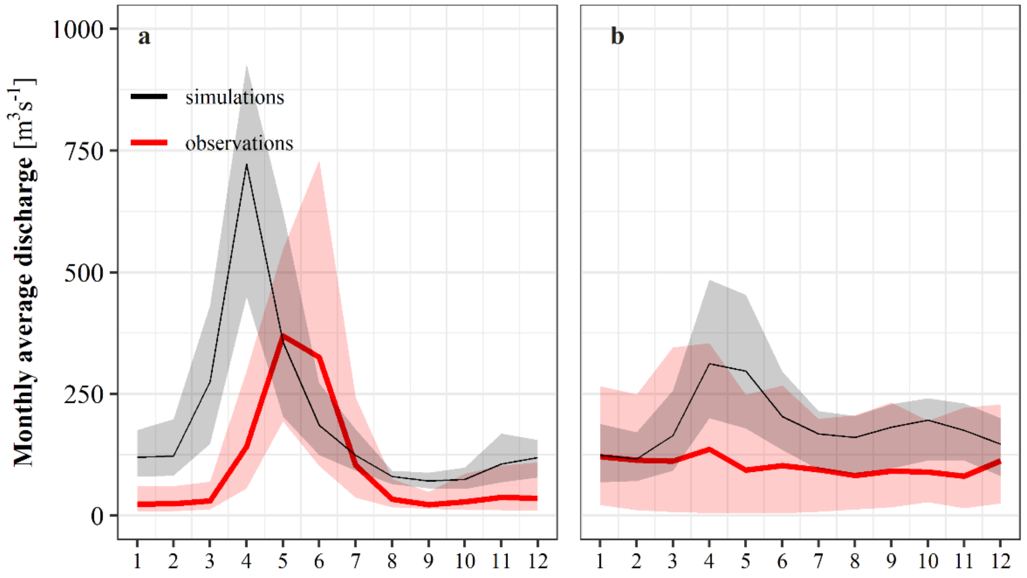

Supplementary Figure 19: Observed and simulated monthly discharges downstream of Hungry Horse dam (US). (**a**) before 1952 and (**b**) after 1952 when the dam entered active operation. The color bands show the 95th intervals associated with the observed (red) and simulated (black) monthly average discharge.

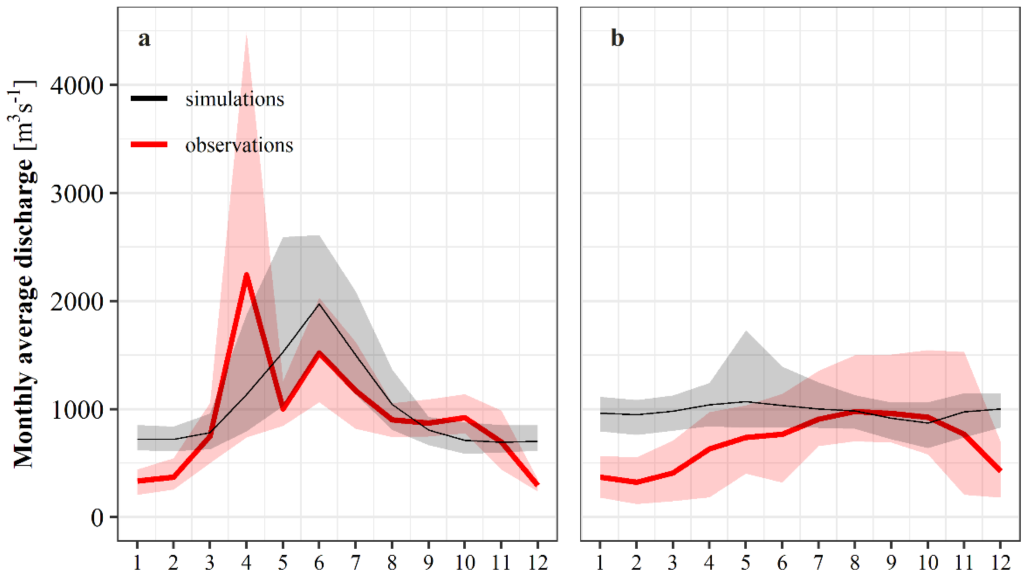

Supplementary Figure 20: Observed and simulated monthly discharges Fort Randall dam (US). (**a**) before 1953 and (**b**) after 1953 when the dam entered active operation. The color bands show the 95th intervals associated with the observed (red) and simulated (black) monthly average discharge.

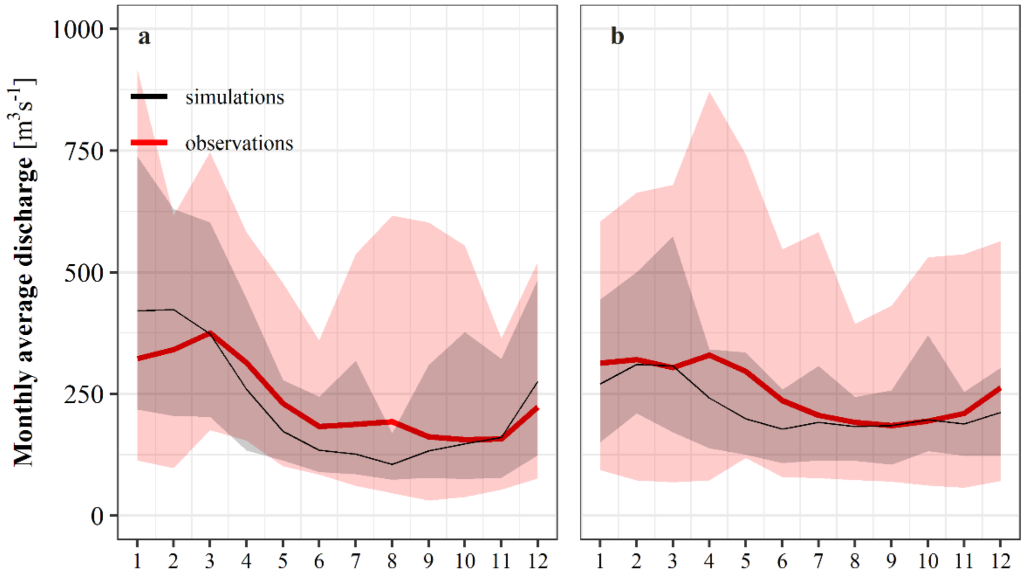

Supplementary Figure 21: Observed and simulated monthly discharges on the Roanoke River (US) below major dams. (**a**) before 1955 and (**b**) after 1955 when all dams entered active operation. The color bands show the 95th intervals associated with the observed (red) and simulated (black) monthly average discharge.

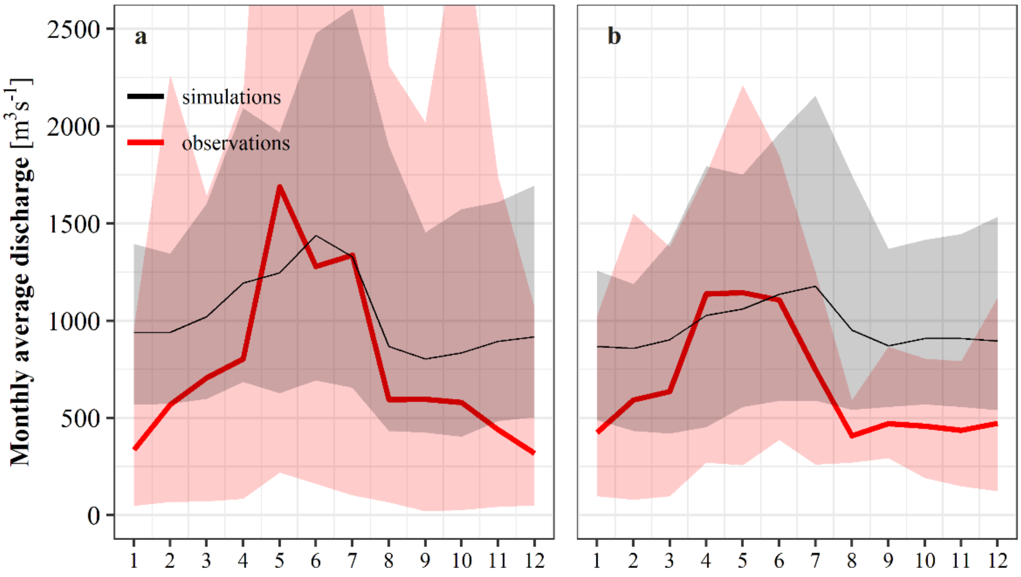

Supplementary Figure 22: Observed and simulated monthly discharges after the Robert S. Kerr dam (US). (**a**) before 1964and (**b**) after 1964 when the dam entered active operation. The color bands show the 95th intervals associated with the observed (red) and simulated (black) monthly average discharge.

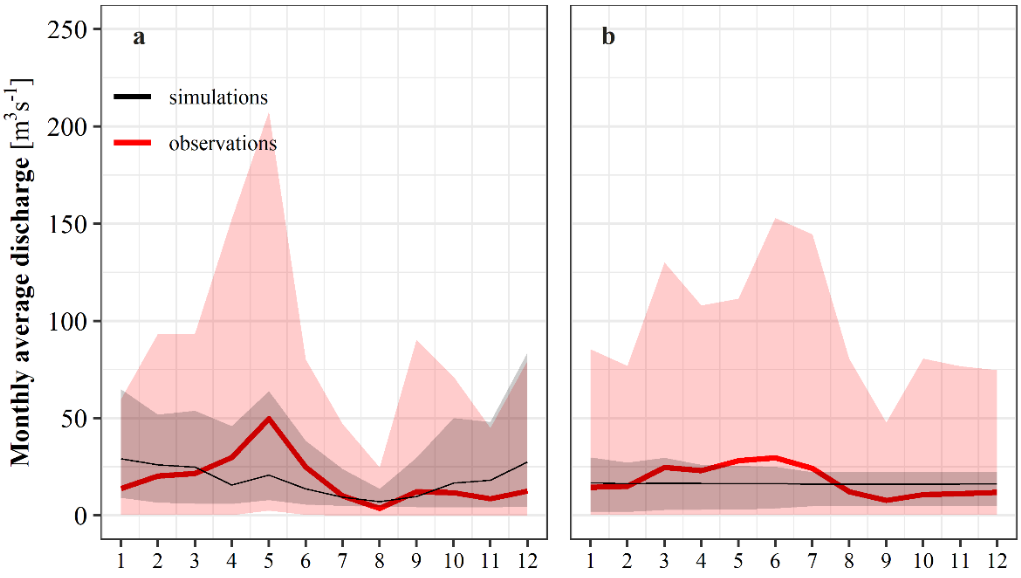

Supplementary Figure 23: Observed and simulated monthly discharges downstream of the Belton dam (US). (**a**) before 1954 and (**b**) after 1954 when the dam entered active operation. The color bands show the 95th intervals associated with the observed (red) and simulated (black) monthly average discharge.

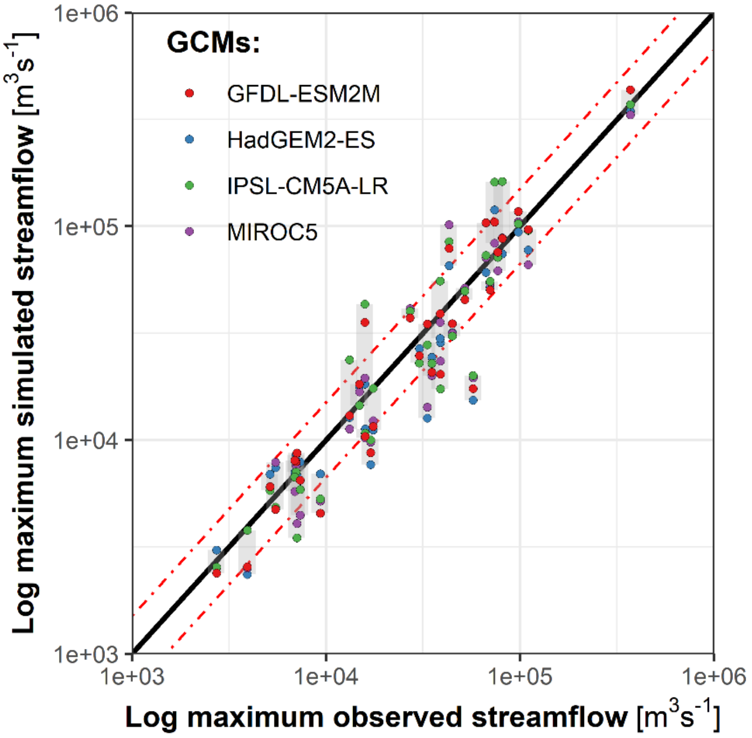

Supplementary Figure 24: Observed and simulated (4 GCMs) maximum daily streamflow in 33 catchments. Note: The shaded grey areas represent the uncertainty in maximum streamflows across all GCMs for a given basin. The 1:1 identity line is represented by the black plain line. The two red dash lines indicate the ±50% range.

Thresholds	30 $m^3 s^{-1}$		150 $m^3 s^{-1}$		300 $m^3 s^{-1}$	
	RCP2.6	RCP6.0	RCP2.6	RCP6.0	RCP2.6	RCP6.0
Agreement land area†	53.6%	62.3%	53.5%	62.3%	53.5%	62.3%
Below dams, decrease flooding‡	66.7%	60.3%	65.4%	59.3%	64.5%	58.0%
Below dams, increase flooding‡	7.5%	5.4%	6.7%	4.7%	6.4%	4.2%

Supplementary Table 1: Robustness of the fitting methodology given different thresholds when comparing extreme discharge series for the experiments considering and not considering dams.
† After data filtering (see Method)
‡ Only for consistent grid cells.

			Ref. 4	Estimates[†]	This study	
Population			2005		2010	
Resolution			2.5’	Country	0.005°	
Forcing data			11 GCMs[‡]	-	4 GCMs	
Flood definition			100-year	100-year	100-year	
Dams			no	yes	no	yes
Exposure (million)	20C		5.6±2.3	11.9 (4.0 – 13.8)	9.4 (4.8 – 15.9)	-
	21C	RCP2.6	23±7	-	31.5 (18.7 – 62.2)	30.1 (18.0 – 59.1)
		RCP4.5	38±11	-	-	-
		RCP6.0	43±16	-	55.2 (31.5 – 83.3)	52.9 (30.7 – 80.1)
		RCP8.0	77±22	-	-	-

Supplementary Table 2: Average population (in million) exposed to river flood in the 20st century (1990–2005) and late 21st century (2070–2100) across studies. As an indication of variability, the standard deviation is given for reference 4 while the first and third quartiles (in millions) are given for the estimate (from Dartmouth Flood Observatory) and this study predictions.

[†] Database from Dartmouth Flood Observatory (http://floodobservatory.colorado.edu/). The number of people displaced (either left homeless after the flood or evacuated during the flood) is shown, only events of severity class 2 were considered (floods with a recurrence interval equal and greater than 100 years).

[‡] For RCP2.6 and RCP6.0, the ensemble consisted of 8 and 5 models, respectively.

No.	River, creek, or location	Dam(s) upstream	Finish year	Drainage area (km^2)	Capacity (10^7 m^3)
1	Nakhon Shawan	Bhumibol, Sirikit	1964 and 1974	39,530	2,300[†]
2	Colorado	Glen Canyon	1963	108,355	369
3	White	Bull Shoals	1951	23,400	710
4	Chattahoochee	West point	1974	3,380	75
5	Iowa	Coralville	1958	3,115	52
6	Sabine	Iron Bridge	1960	8,229	205
7	South Fork Flathead	Hungry Horse	1952	1,640	443
8	Missouri	Fort Randall	1953	322,800	700
9	Roanoke	Kerr, Roanoke, Gaston	1955	8,400	415
10	Arkansas	Robert S. Kerr	1964	74,460	54
11	Leon	Belton	1954	3,560	231

Supplementary Table 3: Locations where monthly streamflow produced by the coupled model was assessed before and after dam(s) construction. All input data for the US site were accessed from the USGS website.

[†] Cumulative capacity

Supplementary References:

1. Hanasaki, N., Kanae, S. & Oki, T. A reservoir operation scheme for global river routing models. *Journal of Hydrology* **327**, 22–41 (2006).
2. Rougé, C. *et al.* Coordination and Control: Limits in Standard Representations of Multi-Reservoir Operations in Hydrological Modeling. *Hydrology and Earth System Sciences Discussions* **2019**, 1–37 (2019).
3. Shin, S., Pokhrel, Y. & Miguez-Macho, G. High-Resolution Modeling of Reservoir Release and Storage Dynamics at the Continental Scale. *Water Resources Research* **55**, 787–810 (2019).
4. Yin, X.-A., Yang, Z.-F. & Petts, G. E. Reservoir operating rules to sustain environmental flows in regulated rivers. *Water Resources Research* **47**, (2011).
5. Mateo, C. M. *et al.* Assessing the impacts of reservoir operation to floodplain inundation by combining hydrological, reservoir management, and hydrodynamic models. *Water Resources Research* **50**, 7245–7266 (2014).
6. Mei, X., Van Gelder, P. H. A. J. M., Dai, Z. & Tang, Z. Impact of dams on flood occurrence of selected rivers in the United States. *Frontiers of Earth Science* **11**, 268–282 (2017).
7. O'Connor, J., E. & Costa, J., E. The World's Largest Floods, Past and Present: Their Causes and Magnitudes. (2004).
8. Yamazaki, D., Kanae, S., Kim, H. & Oki, T. A physically based description of floodplain inundation dynamics in a global river routing model. *Water Resources Research* **47**, (2011).
9. Mueller, E. R. *et al.* Geomorphic change and sediment transport during a small artificial flood in a transformed post-dam delta: The Colorado River delta, United States and Mexico. *Ecological Engineering* **106**, 757–775 (2017).
10. Komori, D. *et al.* Characteristics of the 2011 Chao Phraya River flood in Central Thailand. *Hydrological Research Letters* **6**, 41–46 (2012).
11. Ehsani, N., Vörösmarty, C. J., Fekete, B. M. & Stakhiv, E. Z. Reservoir operations under climate change: Storage capacity options to mitigate risk. *Journal of Hydrology* **555**, 435–446 (2017).
12. FEMA. Identifying High hazard Dam Risk in the United States.
13. Ho, M. *et al.* The future role of dams in the United States of America. *Water Resources Research* **53**, 982–998 (2017).
14. Lempérière, F. Dams and Floods. *Engineering* **3**, 144–149 (2017).
15. May, P., J. & Williams, W. *Disaster Policy Implementation, Managing Programs under Shared Governance*. (Springer, Boston, MA, 1986).
16. Latrubesse, E. M. *et al.* Dam failure and a catastrophic flood in the Mekong basin (Bolaven Plateau), southern Laos, 2018. *Geomorphology* **362**, 107221 (2020).
17. Alcrudo, F. & Mulet, J. Description of the Tous Dam break case study (Spain). *Journal of Hydraulic Research* **45**, 45–57 (2007).
18. Dai, F. C., Lee, C. F., Deng, J. H. & Tham, L. G. The 1786 earthquake-triggered landslide dam and subsequent dam-break flood on the Dadu River, southwestern China — Reply. *Geomorphology* **73**, 277–278 (2006).
19. Hollins, X. L., Eisenberg, A. D. & Seager, P. T. Risk and Resilience at the Oroville Dam. *Infrastructures* **3**, (2018).
20. Zhang, L. M., Xu, Y. & Jia, J. S. Analysis of earth dam failures: A database approach. *Georisk: Assessment and Management of Risk for Engineered Systems and Geohazards* **3**, 184–189 (2009).
21. Zhang, L., Peng, M., Chang, D. & Xu, Y. Statistical Analysis of Failures of Concrete Dams. in *Dam Failure Mechanisms and Risk Assessment* 53–56 (John Wiley & Sons, Ltd, 2016). doi:10.1002/9781118558522.ch4.
22. FitzHugh, T. W. & Vogel, R. M. The impact of dams on flood flows in the United States. *River Research and Applications* **27**, 1192–1215 (2011).
23. Zajac, Z. *et al.* The impact of lake and reservoir parameterization on global streamflow simulation. *Journal of Hydrology* **548**, 552–568 (2017).
24. Zhao, G., Bates, P. & Neal, J. The Impact of Dams on Design Floods in the Conterminous US. *Water Resources Research* **56**, e2019WR025380 (2020).
25. Hirabayashi, Y. *et al.* Global flood risk under climate change. *Nature Climate Change* **3**, 816 (2013).

26. Jongman, B., Ward, P. J. & Aerts, J. C. J. H. Global exposure to river and coastal flooding: Long term trends and changes. *Global Environmental Change* **22**, 823–835 (2012).
27. Jonkman, S. N. Global Perspectives on Loss of Human Life Caused by Floods. *Natural Hazards* **34**, 151–175 (2005).
28. Alfieri, L. *et al.* Global projections of river flood risk in a warmer world. *Earth's Future* **5**, 171–182 (2017).
29. Dottori, F. *et al.* Increased human and economic losses from river flooding with anthropogenic warming. *Nature Climate Change* **8**, 781–786 (2018).
30. Jongman, B. *et al.* Declining vulnerability to river floods and the global benefits of adaptation. *Proceedings of the National Academy of Sciences* **112**, E2271–E2280 (2015).
31. Arnell, N. W. & Gosling, S. N. The impacts of climate change on river flood risk at the global scale. *Climatic Change* **134**, 387–401 (2016).
32. KC, S. & Lutz, W. The human core of the shared socioeconomic pathways: Population scenarios by age, sex and level of education for all countries to 2100. *Global Environmental Change* **42**, 181–192 (2017).
33. Jones, B. & O'Neill, B. C. Spatially explicit global population scenarios consistent with the Shared Socioeconomic Pathways. *Environmental Research Letters* **11**, 084003 (2016).
34. Vollset, S. E. *et al.* Fertility, mortality, migration, and population scenarios for 195 countries and territories from 2017 to 2100: a forecasting analysis for the Global Burden of Disease Study. *The Lancet* doi:10.1016/S0140-6736(20)30677-2.
